# Draft Genome Sequence of Strain ATCC 33958, Reported To Be *Elizabethkingia miricola*

**DOI:** 10.1128/genomeA.00828-15

**Published:** 2015-07-23

**Authors:** Stephanie A. Matyi, Peter R. Hoyt, Patricia Ayoubi-Canaan, Nabeeh A. Hasan, John E. Gustafson

**Affiliations:** a Department of Biochemistry and Molecular Biology, Oklahoma State University, Stillwater, Oklahoma, USA; b Integrated Center for Genes, Environment and Health, National Jewish Health, Denver, Colorado, USA; c Computational Bioscience Program, University of Colorado Denver, School of Medicine, Aurora, Colorado, USA

## Abstract

We report the draft genome of *Elizabethkingia* strain ATCC 33958, which has been classified as *Elizabethkingia miricola*. Similar to other *Elizabethkingia* species, the ATCC 33958 draft genome contains numerous β-lactamase genes. ATCC 33958 also harbors a urease gene cluster which supports classification as *E. miricola*.

## GENOME ANNOUNCEMENT

The Gram-negative genus *Elizabethkingia* consists of *Elizabethkingia meningoseptica* (1–4), *Elizabethkingia anophelis* (5–7), *Elizabethkingia miricola* (3, 8), and *Elizabethkingia endophytica* (9). ATCC 33958 demonstrated 84% DNA-DNA hybridization with the *E. miricola* type strain isolated from condensation in the Russian space laboratory Mir (3, 8). Recent reports of *Elizabethkingia* draft genomes are clinically important since they support the classification of species within this genus. We now report the draft genome of ATCC 33958.

DNA was isolated from an ATCC 33958 brain heart infusion broth culture and then sequenced with a Roche 454 GS Junior. The 429,384 reads were then assembled with the Roche GS De Novo assembler (v2.7) and uploaded to the RAST server for annotation (10). The ATCC 33958 draft genome was 4,578,109 bp (75 contigs, 35.8% GC content) in length and contained 4,421 predicted coding sequences, including 46 tRNA and 3 rRNA genes.

Unlike other *Elizabethkingia* species, *E. miricola* readily hydrolyzes urea (3, 7, 8). RAST analysis revealed the presence of a urease gene cluster (*ureABCEFGD*) in ATCC 33958, which was not found in the *E. meningoseptica* (1) or *E. anophelis* (6) draft genomes.

*Elizabethkingia* species express a multiple antimicrobial resistance phenotype and are resistant to the action of many antimicrobials (11–13). In general, a single β-lactamase gene allows a bacterial pathogen to resist the action of β-lactams and/or related antimicrobials (14, 15). *E. meningoseptica* was the first bacterial pathogen reported to harbor three active β-lactamase genes, which encode a class D serine β-lactamase (16–18), and two unrelated metallo-β-lactamases (16, 19–22). Additionally, *Elizabethkingia* draft genomes have revealed that each species harbors numerous putative β-lactamase genes (1, 6, 23).

Putative β-lactamases identified in the ATCC 33958 RAST annotations were further analyzed with BLASTp (blast.ncbi.nlm.nih.gov) and compared to characterized β-lactamases at http://www.lahey.org/Studies/ and the BRENDA database (http://www.brenda-enzymes.org/), and β-lactamase domains were also identified by using Pfam analysis (http://pfam.sanger.ac.uk). From these analyses we surmised the presence of at least 12 putative β-lactamase genes within the ATCC 33958 draft genome located on 9 contigs. Of these putative β-lactamase genes, 4 demonstrated strong amino acid homologies (42.9% to 98.8% amino acid identity) along the entire length of 4 phenotypically characterized β-lactamases (17, 20, 24, 25). Comparison of the putative ATCC 33958 β-lactamases to one another revealed that only 2 demonstrated significant amino acid identity to each other (42%). It is known that chromosomally encoded β-lactamase genes can be induced by β-lactams and play a role in β-lactam resistance (26). The number and dissimilarity of the β-lactamases within ATCC 33958 suggest that these proteins may contribute to function(s) other than β-lactamase activity. In *Escherichia coli*, for instance, chromosomally encoded β-lactamases display penicillin-binding protein characteristics and play a role in peptidoglycan metabolism (27). The cloning of ATCC 33958 β-lactamase genes will determine if these genes do indeed encode proteins with β-lactamase activity.

### Nucleotide sequence accession numbers.

This whole genome shotgun project has been deposited at DDBJ/EMBL/GenBank under the accession number JRFN00000000. The version described in this paper is version JRFN01000000 for ATCC 33958.
